# Chemical Constituents of the Mexican Mistletoe (*Psittacanthus calyculatus*)

**DOI:** 10.3390/molecules16119397

**Published:** 2011-11-09

**Authors:** Bah Moustapha, Gutiérrez-Avella Dora Marina, Fuentes-Ordaz Raúl, Castañeda-Moreno Raquel, Martínez Mahinda

**Affiliations:** 1 Facultad de Química, Universidad Autónoma de Querétaro, Centro Universitario, Cerro de las Campanas, Querétaro 76010, Mexico; Email: domagu@uaq.mx (G.-A.D.M.); raquelito_cm@hotmail.com (C.-M.R.); 2 Facultad de Ciencias Naturales, Universidad Autónoma de Querétaro, Campus Juriquilla, Querétaro 76230, Mexico; Email: rfuentes@cideteq.mx (F.-O.R.); mahinda@uaq.mx (M.M.)

**Keywords:** *Psittacanthus calyculatus*, isorhamnetin bioside, quercetin bioside, *trans*-4-hydroxy-*N*-methylproline, NMR

## Abstract

A phytochemical study of the methanol-soluble fraction of an aqueous extract of a sample of *Psittacanthus calyculatus* collected from the host plant *Prosopsis laevigata *(Smooth Mesquite) using several techniques, including co-chromatography coupled with UV detection, chromatographic purifications and IR, NMR and MS studies, resulted in the identification of gallic acid, two flavonol-3-biosides and the nonprotein amino acid *N*-methyl-*trans*-4-hydroxy-L-proline.

## 1. Introduction

*Psittacanthus calyculatus* (DC.) G. Don (Loranthaceae) is widely used in Mexican traditional medicine for the treatment of cardiovascular diseases, nowadays the leading cause of deaths in Mexico. It is a semi-parasitic plant that grows on a great variety of harvestable plants, wild or cultivated, belonging to the Rutaceae, woody Leguminosae (Fabaceae), Conniferae, Fagaceae, Myrtaceae (*Eucaliptus*), and Pinaceae families, many of which are fruit trees or timber-yielding plants. In Mexico, it is literally most commonly known as “the plants’cancer” and “true mistletoe”. It is seen by farmers as a very harmful plant because it is associated with major crop losses and sometimes with the death of the host plants. However, it is used in folk medicine as an antiseptic and as a treatment for hypertension [1], as well as for alopecia [2]. In a pharmacological evaluation of the activity of the aqueous extract of a plant sample collected from *Prosopsis laevigata *L. (Fabaceae) on isolated rat aorta, our group previously found that it produces a greater vascular vasodilatory effect than that of the classic vasorelaxant acetylcholine [3]. This observation essentially matches another previous report [4]. However, despite its very promising antihypertensive potential, and apart from a preliminary analysis of a few flavonoids in which hesperitin, quercetin and (+)-catechin were identified [3], its phytochemical contents have not been fully documented. Therefore, we report here the identification of two flavonoid biosides, gallic acid, and the alkaloid *N*-methyl-*trans*-4-hydroxy-L-proline in the methanol soluble fraction of an aqueous extract of the plant.

## 2. Results and Discussion

**Figure 1:**
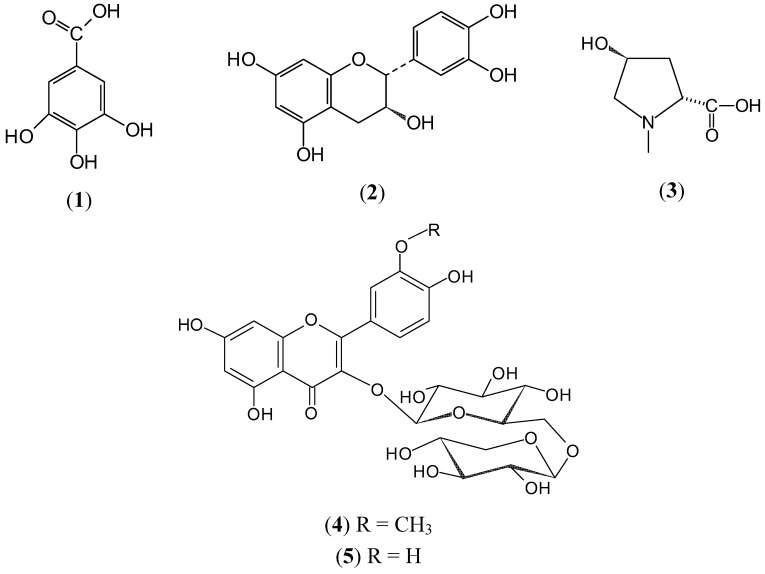
Compounds identified in the MeOH-soluble fraction of the aqueous extract from a sample of *P. calyculatus*.

On account of the important roles phenolic compounds play in promoting health benefits, particularly on the cardiovascular system, the presence of five phenolic acids (gallic, protocatechuic, caffeic, *p*-coumaric, and rosmarinic) and ten flavonoids (hesperidin, rutin, myricetin, luteolin, (+)-catechin, quercetin, apigenin, naringenin, hesperetin, and kaempferol) commonly found in plants was investigated in both non hydrolyzed and hydrolyzed aqueous and methanol extracts by the use of an HPLC apparatus coupled with a UV detector. Retention times and co-chromatography were used to assess the correct identification of the phenolic compounds, and the quantification of those identified was achieved using calibration curves. Of all the phenolic standards investigated, besides (+)-catechin, hesperitin, rutin, and quercetin, already reported [3], only gallic acid (**1**) was identified and then quantified in the aqueous extract. The methanol extract contained none of the standards used. Gallic acid was found to be present mainly in the free form, at a level of 26.6 mg/g of dry plant material (DPM), while an additional low quantity (0.7 mg/g DPM) was obtained after acid hydrolysis. (+)-Catechin (**2**) gave a particularly high yield (59.3 mg/g DPM). As evidenced by these results, most of the secondary metabolites of the aqueous extract are unknown. For this reason, a conventional phytochemical study involving chromatographic analyses and purifications, and spectroscopic identification was undertaken. Column chromatography (CC) on Sephadex LH-20 of the methanol-soluble fraction of the aqueous extract followed by HPLC purification led to the isolation of *trans* 4-hydroxy-*N*-methylproline (**3**). In addition, two major flavonol glycosides, isorhamnetin 3-*O*-β-D-xylopyranosyl (l→6)-β-D-glucopyranoside (**4**) and quercetin-3-*O*-β-D-xylopyranosyl (1→6)-β-D-glucopyranoside (**5**) (Figure 1) were also purified from another portion of the methanol fraction, using successive Si-gel CC followed by reversed-phase HPLC. The structures of these three compounds were determined by spectroscopic studies. Many other minor compounds were isolated during the same process. However, their yields were insufficient for further purification and subsequent identification by NMR spectroscopy. Although the NMR data of the compounds **3**–**5** in other solvents are already reported [5,6,7], we report here those now obtained in CD_3_OD for compound **3** (see Experimental), and in C_5_D_5_N for compounds **4** and **5** (Table 1).

**Table 1:** ^1^H (500 MHz) and ^13^C-NMR (125.7 MHz) spectral data of compounds **4** and **5** (in C_5_D_5_N).

4			5		
	δ_H_ (*J* in Hz)	δ_C_		δ_H_ (*J* in Hz)	δ_C_
2		157.6	2		158.2
3		134.9	3		135.2
4		178.6	4		178.6
5		162.8	5		162.7
6	6.66 (δ, 2.0)	99.9	6	6.63 (δ, 2.0)	99.8
7		166.0	7		165.9
8	6.73 (δ, 2.0)	94.7	8	6.62 (δ, 2.0)	94.6
9		157.7	9		157.7
10		105.6	10		105.4
1′		122.1	1′		122.5
2′	8.50 (δ, 2.0)	114.4	2′	8.37 (δ, 2.0)	117.9
3′		148.1	3′		146.7
4′		151.3	4′		150.7
5′	7.27 (δ, 8.0)	116.3	5′	7.30 (δ, 8.0)	116.3
6′	7.88 (δδ, 8.0, 2.0)	123.4	6′	8.10 (δδ, 8.0, 2.0)	123.0
CH3O	3.95 (σ)	56.3			
Gla-1	6.26 (δ, 7.5)	103.8	Gla-1	6.03 (δ, 7.5)	104.2
Gl-2	4.28 (dd, 9.0, 7.5)	76.1	Gl-2	4.21 (dd, 9.0, 7.5)	75.9
Gl-3	4.31 (δδ, 9.0, 9.0)	78.5	Gl-3	4.22 (δδ, 9.5, 9.0)	78.5
Gl-4	4.21 (δδ, 10.0, 9.0)	71.5	Gl-4	4.06 (δδ, 9.5, 9.5)	71.5
Gl-5	4.07 (m)	77.7	Gl-5	4.09 (m)	77.7
Gl-6	4.68 (dd, 11.2, 2.0)	69.7	Gl-6	4.68 (dd, 11.0, 1.5)	69.7
	4.19 (m)			4.14 (m)	
Xy ^a^-1	4.76 (d, 7.5)	105.6	Xy ^a^-1	4.82 (d, 7.0)	105.6
Xy-2	3.84 (dd, 8.5, 7.5)	74.7	Xy-2	3.88 (dd, 8.5, 7.0)	74.7
Xy-3	3.96 (dd, 8.5, 8.5)	78.0	Xy-3	4.00 (dd, 8.5, 8.5)	77.6
Xy-4	4.06 (ddd, 10.0, 8.5, 5.0)	70.9	Xy-4	4.06 (m)	71.0
Xy-5	4.18 (m)	66.8	Xy-5	4.19 (m)	66.7
	3.48 (dd, 11.2, 10.2)			3.55 dd (11.0, 10.0)	

^a^ Gl = glucose, Xy = xylose.

Gallic acid was already reported in *P. cucullaris* [8]), and (+)-catechin along with tyramine in *P. cuneifolius* [9,10]; however, to the best of our knowledge, this is the first report of the occurrence of compounds **3**–**5** in the genus *Psittachathus*. Good anti-inflammatory activity has been recognized for gallic acid [11,12], while quercetin and isorhamnetin, which can be released from their glycosides in physiological conditions, have been shown to produce *in vitro* vasodilatory effects [13,14] and cardioprotective effects in rats [15,16,17]. Furthermore, recent studies have found a reduction in blood pressure when hypertensive animals and humans are given food supplemented with quercetin [18], so it is plausible to assert that the two flavonol glycosides **4** and **5**, besides the other phenolics found in the aqueous extract of these plant species, contribute greatly to its hypotensive effect. The effect compound **3** might have on any part of the cardiovascular system is as yet unreported.

## 3. Experimental

### 3.1. General

HPLC separations were conducted using a Waters apparatus (Millipore Corp., Waters Chromatography Division, Milford, MA, USA), composed of a 600E multisolvent delivery system equipped with a 486 tunable UV detector. Control of the equipment, data acquisition, processing, and management of the chromatographic information were performed by the Empower 2 software (Waters). NMR spectra were run on Varian Inova NMR spectrometers equipped with 5 mm ^1^H and ^13^C probes and operating respectively at 400 and 100 MHz for compound **3**, and 500 and 125.7 MHz, respectively, for compounds **4** and **5**, with TMS as the internal standard. Mass and IR spectra were obtained on a VG 7070E and Perkin Elmer 283B spectrometers, respectively.

### 3.2. Plant Material

A whole *P. calyculatus* sample was collected from *Prosopsis laevigata*, its most common host plant in Querétaro and in all central Mexico. Collection was made at the botanical garden of the Universidad Autónoma de Querétaro. The sample was then dried in an oven set at 45 °C for 10 days. The dried plant was milled before extraction.

### 3.3. Extraction

The milled dry plant material (approximately 2 kg) was extracted at 60 °C by stirring it together with 2% aqueous ethanol (v/v) (total volume: 5 l) for three days in total darkness. The extract was then freeze-dried.

### 3.4. Isolation and Characterization of trans-4-Hydroxy-N-methylproline (**3**)

The dry extract was mixed with MeOH by shaking. The liquid layer was evaporated until dry under reduced pressure. A portion of this MeOH extract (0.5764 g) was fractionated in a Sephadex LH-20 column chromatography eluted with this solvent. Seven fractions of 500 mL were collected. Fraction 3 (52.25 mg) was separated isocratically by HPLC using a C-18 column (Symmetry: 300 mm × 19 mm i.d.; mobile phase: H_2_O-CH_3_CN 6:4; flow rate: 3 mL/min; UV detection at 254 nm). The most abundant of the five collected compounds (22.42 mg, *t*_R_ 26.39 min) was purified by recycling HPLC to give 3.74 mg of an amorphous white powder that turned slightly brown after a few weeks. Its structural characterization was achieved through its IR, MS, and NMR data. There were characteristic IR (film) bands at 3375 cm^−1^, 2926 cm^−1^ and 2853 cm^−1^, 1625 cm^−1^ (υ_as_COO^-^), 1400 cm^−1^ (υ_s_COO^-^), and 1073 cm^−1^ (C-N). EIMS gave peaks at m/z 145 (M^+^, 5%), 100 (100%), 82, 42 and 18. ^1^H-NMR (400 MHz, CD_3_OD) showed the following signals: δ 4.48 (m, H-4), 4.05 (dd, *J* = 10.6, 7.6 Hz, H-2), 3.78 (dd, *J* = 12.8, 4.4 Hz, H-5α), 3.05 (ddd, *J* = 12.4, 2.0, 2.0 Hz, H-5β), 2.98 (s, CH_3_-N), 2.43 (dddd, *J* = 13.6, 7.6, 2.0, 2.0 Hz, H-3α), 2.15 (ddd, *J* = 13.8, 11.0 and 4.8 Hz, H-3β), and ^13^C-NMR (100 MHz, CD_3_OD) gave signals at δ 172.8 (C=O), 71.9 (C-2), 70.9 (C-4), 64.1 (C-5), 44.0 (CH_3_-N) and 40.3 (C-3).

### 3.5. Isolation of Isorhamnetin 3-O-β-D-xylopyranosyl (1→6)-β-D-glucopyranoside (**4**) and Quercetin 3-O-β-D-xylopyranosyl (1→6)-β-D-glucopyranoside (**5**)

A second portion (5.25 g) of the methanol extract was subjected to open CC on Si-gel (J.T.Baker, 60–200 mesh), employing a gradient elution with AcOEt (solvent A) and MeOH (solvent B), from 100% to 0% A. A total of 71 fractions were collected. Fractions 25–34, eluted with AcOEt-MeOH (95:5), were combined and analyzed by HPLC. Scaling up to semi-preparative HPLC using an XDB-C18 column (ZORBAX) (21.2 × 150 mm; 5 mm; 65 min of linear gradient elution with aqueous AcOH 0.0125 N from 100% to 60%, and CH_3_CN; flow rate: 4 mL/min; detection at 280 nm) yielded the two main flavonol glycosides **4** (8.1 mg; *t*_R_ 49.55 min) and **5** (6.9 mg, *t*_R_ 43.26 min) as slightly yellow amorphous powders. NMR data: see Table 1.

## 4. Conclusions

Although the vasodilatory effects of the aqueous extract of this medicinal plant was previously described, this is the first phytochemical study reporting the isolation of some of its constituents, most of which could contribute to its hypotensive effect.
